# *Lepidium sativum* as candidate against excitotoxicity in retinal ganglion cells

**DOI:** 10.1515/tnsci-2020-0174

**Published:** 2021-06-04

**Authors:** Abeer Al-Dbass, Musarat Amina, Nawal M. Al Musayeib, Amira A. El-Anssary, Ramesa Shafi Bhat, Rania Fahmy, Majd M. Alhamdan, Afaf El-Ansary

**Affiliations:** Biochemistry Department, College of Sciences, King Saud University, Riyadh, Saudi Arabia; Department of Pharmacognosy, Pharmacy College, King Saud University, Riyadh, Saudi Arabia; Department of Pharmacognosy, National Research Centre, Giza, Egypt; Department of Ophthalmology, Faculty of Medicine, Cairo University, Cairo, Egypt; Department of Optometry, College of Applied Medical Sciences, King Saud University, Riyadh, Saudi Arabia; Pharmacy College, King Saud University, Riyadh, Saudi Arabia; Central Laboratory, Female Centre for Scientific and Medical Studies, King Saud University, Riyadh, Saudi Arabia

**Keywords:** *Lepidium sativum*, glutamate excitotoxicity, retinal ganglion cell, cell viability, COMET assay

## Abstract

Glutamate excitotoxicity is considered one of the major causes of retinal ganglion cell death in many retinal diseases. Retinal ganglion cell degeneration causes severe blindness since visual signals from the eye to the brain are conducted only through retinal ganglion cells. Objective: We aimed to explore the potential ameliorative effects of *L. sativum* against glutamate excitotoxicity-induced retinal ganglion cell damage. Methods: Pure retinal ganglion cells were divided into a control group (untreated); *L. sativum*-treated groups in which retinal ganglion cells were treated with 5, 10, 50, or 100 µg/mL *L. sativum* seed extract for 2 h; glutamate-treated groups in which cells were treated with 5, 10, 50, or 100 µM glutamate for 48 h; and *L. sativum*/glutamate groups [pretreatment with *L. sativum* for 2 h (50 or 100 µg/mL) before glutamate treatment at 100 µM for 48 h]. Cell damage was assessed by comet assay and cell viability was by MTT test. Results: Tailed DNA, tail length, and tail moment of the 50 and 100 mM glutamate-treated groups were significantly greater than those of the blank control group, while the *L. sativum*-treated groups demonstrated nonsignificantly different tailed DNA, tail length, and tail moment compared with the blank control group, but significantly lower values compared with the glutamate-treated groups. Conclusion: *L. sativum* ameliorated the cell viability in retinal ganglion cells after high-concentration glutamate exposure. *L. sativum* seed extracts were efficient anti-excitotoxic and antioxidant agent that might improve the clinical presentation of many neurological disorders.

## 1 Introduction

Retinal ganglion cells (RGCs) are the sole efferent nerve cells that transmit visual impulses from the retinal bipolar and amacrine cells to the cortical visual centers (the lateral geniculate nucleus and the superior colliculus). They are categorized into 40 subtypes according to their morphology and functions. The visual information is chemical messages received by transmembrane receptors on RGCs and transformed into electrical signals that travel along the optic nerve to the visual cortex [1,2].

Many retinal pathologies result in RGC degeneration with subsequent vision loss. Increasing evidence has indicated that RGCs are vulnerable to various insults in a type-specific manner [3,4]

Glutamate excitotoxicity is considered a pathological process in which nerve cells are destroyed by extreme excitation of glutamate receptors [*N*-methyl-d-aspartate (NMDA) receptors]. It plays a significant role in the necrosis of neurons in many neurological disorders [5]. Glutamate receptors are expressed by all types of retinal RGCs [6] and NMDA excitotoxicity is believed to result in RGC death in many retinal pathologies [7].

In various neurodegenerative diseases, optic nerve degeneration has been confirmed as part of the pathological process of central nervous system degeneration. Retinal optical coherence tomography revealed thinning of RGCs and the optic nerve-forming axons in neurodevelopmental and neurodegenerative diseases such as autism spectrum disorders, Alzheimer’s disease [8], Parkinson’s disease [9], Huntington’s disease [10], and multiple system atrophy [11].

Medicinal plants have played a significant role in providing humans with formulations to cure and prevent various diseases and ailments. Phytotherapy is now recognized as a promising field in healthcare and serves as a supportive approach for the treatment of various common ailments [12].

Different neurological disorders such as trauma, ischemia, stroke, and epilepsy as well as neurodevelopmental disorders such as autism, despite having diverse primary causes of onset, share a common final damaging pathway known as excitotoxicity [13].

The concentration of glutamate in the synaptic cleft is a critical factor of endogenous excitotoxicity because the synaptic cleft is the site where glutamate interacts with postsynaptic receptors. Excitotoxic neuronal death is thought to be a consequence of intracellular Ca^2+^ influx. Under normal physiological conditions, the concentration of glutamate within the synaptic cleft increases very briefly to 1–2 mM as it is released from presynaptic vesicles by exocytosis. Synaptic transmission is terminated by the combination of reuptake by glutamate transporters, glutamate diffusion out of the synaptic cleft, and receptor di-sensitization [14,15]. Increasing evidence indicates that glutamate excitotoxicity and/or oxidative stress is associated with mitochondrial DNA (mtDNA) damage-related mitochondrial dysfunction in retinal neurodegeneration [16,17,18,19].

The currently available drug therapies for neurodegenerative diseases are palliative, with limited effectiveness and adverse side effects [20,21,22]. The major challenge for researchers is to develop a therapy that addresses the underlying cause/mechanism of degeneration with improved effectiveness and the least side effects. Therapeutic interventions that modify the progression of neurodegeneration may prove useful and plant-based interventions offer various possibilities to modify disease progression and symptoms [22].

Against this background, *Lepidium sativum* Linn. from the family Brassicaceae was selected on the basis of its excellent chemical, pharmacological, and nutritional profiles [23,24]

Several studies have focused on the nutritional and medicinal potential of this plant species, but our study is first of its kind to evaluate the anti-excitotoxicity of *L. sativum.* This species (with the common name garden cress or garden pepperwort) is an annual, edible, fast-growing herb grown throughout India, the United States, Europe, and the Middle East. The seeds and leaves of this plant are rich in numerous bioactive phytochemicals [25,26]. The seed extract of *L. sativum* is used as a medicine in Ayurveda (Indian system of medicine) [25] as well as having multiple applications in Saudi folk medicine, mainly in fracture healing, anti-inflammation, and at the postpartum stage [27]. It also exhibits valuable pharmacological effects, including antioxidant, anticancer, cardiovascular, diuretic [28,29,30,31] anti-inflammatory, analgesic, antipyretic [32,33], antidiabetic, antimicrobial, hypocholesterolemic, antispasmodic, antidiarrheal, and laxative effects [34].

Although a number of medicinal plants are well-known to be a potent storehouse of polyphenolic and flavonoid phytoconstituents, there have been few studies evaluating their anti-excitotoxicity properties and their ability to prevent reactive oxygen species (ROS)-mediated cellular and DNA damage. The Food and Agriculture Organization (FAO) has classified *L. sativum* as an underutilized or neglected crop among ancient crops.

In this context, the aim of the present study was to evaluate the comprehensive ameliorative effects of *L. sativum* as an edible plant herb against glutamate excitotoxicity-induced RGCs as an etiological mechanism of many neurological disorders.

## 2 Materials and methods

### 2.1 Botanical material

*Lepidium sativum* L. (1.0 kg) seeds were purchased from a local market in Riyadh, Saudi Arabia. The collected seeds were washed with tap water, disinfected, rinsed with distilled water, and finally dried under shaded conditions. These dried seeds were ground into a fine powder. The plant species were taxonomically identified and authenticated by Prof. Mohamed Yousef of the Department of Pharmacognosy, College of Pharmacy, King Saud University, Saudi Arabia. Voucher specimens (LP/8619 and MO/8719) were deposited at the herbarium of the department.

### 2.2 Preparation of extracts

The dried, powdered seeds (1.0 kg) of *L. sativum* were extracted rigorously with methanol in a Soxhlet apparatus for 2 days. The combined extract was filtered through double-layered muslin cloth, centrifuged at 10,000 rpm for 5 min, and finally filtered through Whatman No. 1 filter paper to obtain a clear filtrate. The obtained filtrates were separated from the solvent on a wiped film evaporator at 50 ± 5°C under reduced pressure by using a vacuum evaporator. The yield obtained for the extract was 54.24 g. The extract was stored in capped bottles in a refrigerator at 5°C prior to use.

### 2.3 Phytochemical screening of *L. sativum* seeds

Phytochemical analysis of the crude seed extract of *L. sativum* was performed qualitatively to determine the presence of various phytoconstituents such as tannins, flavonoids, triterpenoids, alkaloids, saponins, and anthraquinones as plant constituents using standard phytochemical methods following the work of Evans [35]. Briefly, the presence of tannins in the plant extracts was detected by the formation of green or black precipitate upon treatment with aqueous FeCl_3_. The plant samples were considered positive for flavonoids when a yellow color was precipitated out with 10% lead acetate solution. Triterpenoids were screened by treating plant extracts with acetic anhydride and a few drops of conc. H_2_SO_4_. The formation of a bluish green ring or violet or blue color indicated their presence. The alkaloids were tested by treating the plant extracts with Dragendoff’s reagent, resulting in the formation of turbid precipitate at the bottom of the test tube indicating their presence. The saponins were detected in the plant material when diluted samples upon vigorous shaking resulted in stable froth formation. The plant samples were deemed positive for cardiac glycosides when a reddish-brown color appeared with conc. H_2_SO_4_. Finally, anthraquinones were screened in plant samples by dissolving plant extracts in 1% HCl followed by the addition of NH_4_OH reagent and purified with benzene. The formation of a pink, violet, or red color indicated their presence.

### 2.4 High‐performance liquid chromatography (HPLC) analysis of *L. stavium* seed extract

The HPLC analysis of crude methanolic *L. stavium* seed extract was performed on a Shimadu i-series of liquid chromatograph comprising of a solvent delivery pump Model LC-20AT, controller Model LC-2030, UV visible detector with variable wavelengths Model UV-2600, and autosampler equipped with a photodiode array detector (SPD-M30A, Shimadzu). Separation of flavonoids was performed on Inertsil ODS III analytical column with 250 mm × 4.6 mm dimensions and 5 μm particle size. The analytical column was externally protected by C18 Guard‐Pak cartridge (Waters, Milford). Injection volume was used to be 10 µL. The mobile used were: 0.2% of acetic acid in 45% of Millipore water and 0.2% of acetic acid in methanol and an isocratic program was applied. All the separations were carried out at 20°C temperatures with a 0.8 mL/min flow rate. The peaks were identified on the chromatogram by comparing their retention times and spectra with those of commercial and authentic standard solution. Identification of flavonoid compounds was based on the appearance of the absorption maxima between 275 and 320 nm in the spectrum of the individual peaks.

### 2.5 Preparation of retinal cell suspensions

The retinal tissues were separated from the enucleated eyeballs of newborn Sprague-Dawley rats on postnatal days 1 to 4 and incubated in precooled calcium-free and magnesium-free Earle’s Balanced Salt Solution (EBSS; Gibco, Grand Island, NY) and Hank’s Balanced Salt Solution (Life Technologies, Grand Island, NY) containing 5 mg/mL papain, 0.24 mg/mL l-cysteine, and 10 U/mL DNase І for 30 min. Then, an ovomucoid solution containing 0.1% bovine serum albumin (BSA; Sigma-Aldrich, St. Louis, MO), 0.1% ovomucoid (Sigma-Aldrich), and 1% DNase І (4 mg/mL, Sigma-Aldrich) in minimum essential medium (MEM; Gibco) was subsequently used to fully quench any residual papain activity. After centrifugation at 200×*g* for 10 min, the cells were resuspended in MEM containing 0.5 mg/mL BSA and then the cell suspension was filtered through the mesh filter (pore size 40 μm; BD Falcon, Franklin Lakes, NJ) to yield a single cell suspension. The procedures were conducted at room temperature in a laminar flow hood.


**Ethical approval:** The research related to animals’ use has been complied with all the relevant national regulations and institutional policies for the care and use of animals.

### 2.6 RGC purification: preparation of panning dishes and cell culture dishes/plates

Antibody-coated 10-cm Petri dishes (one dish per eight rats) were prepared for negative or positive selection by adding 15 μL of a rabbit anti-rat macrophage/Thy-1 antibody and 7 mL of 50 mM Tris-HCl (pH 9.5) per dish. The plates were swirled until the surfaces were evenly coated with the antibody-Tris solution. The panning plates were incubated overnight at 4°C. Immediately before use, the plates were rinsed three times with Dulbecco’s PBS (1×; 0.9 mM CaCl_2_, 0.49 mM MgCl_2_·6H_2_O, 137.9 mM NaCl, 2.67 mM KCl, 8.06 mM Na_2_HPO_4_·7H_2_O, 1.47 mM KH_2_PO_4_, pH 7.4; D-PBS; Gibco). Then, 1× poly-d-lysine stock (PDL; Sigma-Aldrich) was added to the cell culture plates (50 μL for the 96-well plates, 100 μL for the 24-well plates, and 500 μL for the six-well plates), and the plates were incubated overnight at room temperature. The plates were rinsed three to four times with sterile H_2_O and aspirated to dryness. Mouse laminin (1 mg/mL) was diluted to a final concentration of 50 μg/mL by adding 10 μL of the laminin stock to 5 mL of Neurobasal medium (Gibco, Grand Island, NY). The diluted laminin solution was mixed well, added to the dried cell culture plates, and incubated in a 37°C incubator for 2 h. The plates were rinsed with D-PBS three times before use. To isolate RGCs with immunopanning (IP), we adapted a protocol from Cold Spring Harbor Protocols.

### 2.7 Cell culture

The purified RGCs were seeded at the desired density on the PDL- and laminin-coated coverslips in 24-well or six-well plates in prewarmed RGC growth medium and maintained in a 37°C cell culture incubator with a humidified atmosphere containing 5% CO_2_ and 95% air. The RGC growth medium was improved from the formulation described in the Cold Spring Harbor Protocols, based on our repeated experiments, and contained Neurobasal medium, BSA (0.1 mg/mL; Sigma), transferrin (0.1 mg/mL; Sigma), progesterone (60 ng/mL; Sigma), putrescine (16 µg/mL; Sigma), selenium (40 ng/mL; Sigma), 3,5,3-triiodothyronine T3 (40 ng/mL; Sigma), thyroxine T4 (40 ng/mL; Sigma), B27 (20 µl/mL; Invitrogen), sodium pyruvate (1 mM; Gibco), glutamine (2 mM; Gibco), *N*-acetyl-l-cysteine (NAC, 5 µg/mL; Sigma), insulin (5 µg/mL; Sigma), forskolin (5 µM; Sigma), brain-derived neurotrophic factor (BDNF, 50 ng/mL; PeproTech, Rocky Hill, NJ), ciliary neurotrophic factor (CNTF, 10 ng/mL; PeproTech), basic fibroblast growth factor (bFGF, 10 ng/mL; PeproTech), and penicillin-streptomycin (100 U/mL; Gibco). Half of the medium was replenished every 3 days. However, although a high level of purity was obtained with FACS, the level of cell survival was low. The causes of this low survival are unclear.

### 2.8 COMET assay

The comet assay was performed in accordance with the procedure established by Singh et al. [36], with some modifications. Briefly, after 24 h of exposure of cells to the tested substance in different Petri dishes (60 × 15 mm; Greiner), the cells were trypsinized (0.1% for 4 min), suspended, homogenized in 1 mL of medium, and centrifuged (10 min at 800 rpm). Subsequently, 600 μL of low-melting agarose (0.8% in PBS) was added to the cell suspension (100 μL). Then, 100 μL of this mixture was transferred to agarose pre-coated slides. The coated slides were immersed in lysis buffer (0.045 M TBE, pH 8.4, containing 2.5% SDS) for 15 min. The slides were placed in an electrophoresis chamber containing the same TBE buffer, but devoid of SDS. The electrophoresis conditions were 2 V/cm for 2 min and 100 mA. Staining was performed using ethidium bromide (20 μg/mL at 4°C; Sigma). Observation was performed when the samples were still humid; the DNA fragment migration patterns of 100 cells for each dose level were evaluated with a fluorescence microscope [with excitation filter 420–490 nm (issue 510 nm)]. The comet tail lengths were measured from the middle of the nucleus to the end of the tail with a 40× increase for the count and measure the size of the comet. For the visualization of DNA damage, observations of ethidium bromide-stained DNA were performed using a fluorescent microscope using public domain software for image analysis based on Comet 5 image analysis software developed by Kinetic Imaging, Ltd. (Liverpool, UK), linked to a CCD camera. DNA damage was measured as tail length (TL = distance of DNA migration from the center of the body of the nuclear core) and tail intensity of DNA (TI = % of genomic DNA that migrated during the electrophoresis from the nuclear core to the tail). By presenting all three parameters together, more information on the extent of DNA damage could be obtained.

### 2.9 *L. sativum* neuroprotective effects on glutamate-induced excitotoxicity

Pure RGCs were used for the current study. They were divided into four (sets of) groups according to the various treatments: control group (untreated); *L. sativum*-treated groups in which RGCs were treated independently with 5, 10, 50, or 100 µg/mL *L. sativum* seed extract for 2 h to test the cytotoxic effects of the extract compared with control-untreated RGCs; glutamate-treated groups (glutamate treatment for 48 h using 5, 10, 50, or 100 µM glutamate); and *L. sativum*/glutamate groups [pretreatment with *L. sativum* for 2 h (50 and 100 µg/mL) before glutamate treatment at 100 µM for 48 h].

### 2.10 Determination of cell viability

All cultured RGCs were cultured in a 96-well plate. After treatment, the cell viability was measured using the MTT test. MTT solution (5 mg/mL) was added to each well (20 µL/well) at a final concentration of 0.5 mg/mL and incubated at 37°C for 4 h. Subsequently, the reaction was terminated by adding 200 µL of DMSO to each well for 15 min. The absorbance of each sample was measured at 490 nm using a microplate reader (Bio-Rad). The results are presented as a percentage of control (untreated cells) or glutamate excitotoxic independently.

### 2.11 *In vitro* antioxidant activity of *L. stavium*

The free radical-scavenging potential of methanolic seed extract of *L. stavium* was measured by applying two *in vitro* assays such as ABTS˙^+^ assay and DPPH˙ assay.

#### 2.11.1 ABTS radical-scavenging activity

ABTS radical-scavenging activity of the *L. stavium* seed extract was performed by following the procedure described by Re et al. [37]. Equal amounts of ABST stocks (5 mL) and 2.45 mM potassium persulfate (5 mL) were mixed and allowed to stand in dark for 16 h at ambient temperature to produce ABTS radical cation (ABTS˙^+^). Prior to use, the solution was diluted with distilled water to obtain 0.700 ± 0.020 absorbance at 734 nm and equilibrated at 30°C. Different concentrations of plant extract were diluted with dimethyl sulfoxide (DMSO) to prepare the sample solution. 5 µL of each sample solution was merged with 195 µL ABTS˙^+^ solution and the reaction mixture was incubated for 6 min at room temperature. Afterwards, the absorbance of the reaction mixture was measured at 734 nm. Butylated hydroxytoluene (BHT) was used as a control and the experiments were performed in triplicates. The ABTS scavenging capacity of plant extract was expressed as IC_50_ (µg/mL) and the inhibition percentage was determined by applying following equation:

(1) \text{ABTS}\hspace{.5em}\text{scavenging}\hspace{.5em}\text{activity}\hspace{.5em}( \% )=\frac{{A}_{0}-{A}_{1}}{{A}_{0}}\times 100]

where *A*_0_ and *A*_1_ are the absorbance of control and sample, respectively.

#### 2.11.2 DPPH radical-scavenging activity

DPPH free radical-scavenging activity of the extract was measured by obeying the method described by Blois [38]. DPPH solution was prepared by dissolving 0.025 g of DPPH in 1 L of methanol. Various concentrations of plant extract were diluted DMSO to obtain the sample solution. 5 µL of sample solution was added in a 96-well plate followed by the addition of 195 µL of DPPH working solution in each well and the mixture was incubated for 20 min at ambient temperature under dark conditions. After 20 min incubation, the absorbance of the reaction mixture was recorded at 515 nm. BHT was applied as a standard. All the tests were carried out in triplicates and inhibition percentage was determined by comparing absorbance values of the test sample with that of control. The DPPH scavenging ability of test sample was expressed as IC_50_ (µg/mL) and the inhibition percentage was calculated by applying equation (1).

### 2.12 Statistical analysis

The data were analyzed using the Statistical Package for the Social Sciences (SPSS, Chicago, IL, USA). The results are presented as mean ± standard deviation (S.D). Comparing between all groups using one-way ANOVA test with multiple comparisons (Dunnett s test) to compare each group with the control group for (Parametric data), and Comparing between all groups using Kruskal–Wallis test, and using Mann–Whitney test to compare each group with the control group for (Nonparametric data). Significance was assigned at the level of *p* < 0.05.

## 3 Results

### 3.1 HPLC-DAD detection of methanolic extract of *L. stavium* seed

The HPLC-DAD chromatograph of extract displayed adjacent peaks of the main flavonoids identified in the methanolic extract of *L. stavium* seed within shorter analysis time. Due to the close similarity in the structures, the separation of the flavonoid constituents was difficult. The separation depends on the percentage of flavonoid constituents in the extract and selection of appropriate mobile phase (methanol, water, acetic acid).

**Figure 1:**
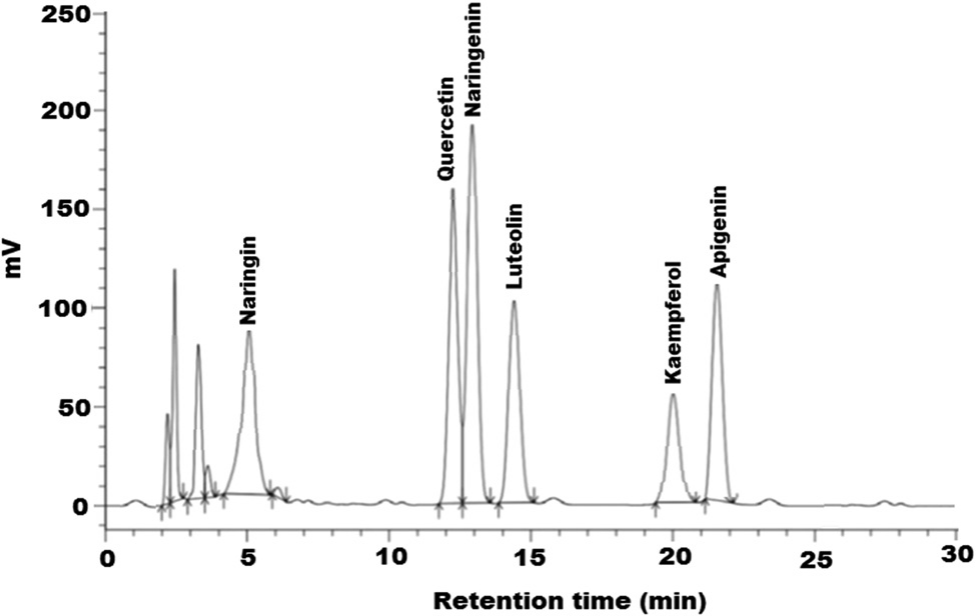
HPLC profile of flavonoids from methanolic *L. sativum* seed extract at 275 nm.

As shown in Figure 1, six major peaks for flavonoids were detected, including naringin, quercetin, naringenin, luteolin, kaempferol, and apigenin in the chromatogram of the seed extract of *L.stavium*. The amount of these components was calculated and presented in Table 1 and shown in Figure 1. Phytochemical screening of crude methanol extracts of *L. sativum* is shown in Table 2.

**Table 1:** Concentration of the identified flavonoids components in the *L. sativum* seed extract

*L. sativum* seed extract					Standard	
Peak number	Identified compounds	Retention time (*t*_R_)	Peak area	Concentration in extract (mg/g)	Retention time (*t*_R_)	Peak area
1	Naringin	5.114	2,781,628	2.62	5.048	8,721,813
2	Quercetin	12.248	4,882,629	10.50	12.241	12,681,712
3	Naringenin	12.816	5,878,472	21.67	12.764	23,456,321
4	Luteolin	14.287	3,183,742	2.82	14.642	12,681,722
5	Kaempferol	20.211	24,516,535	1.67	20.212	17,148,150
6	Apigenin	21.401	528,396	4.35	22.116	2,329,872

**Table 2:** Phytochemical screening of crude methanol extracts of *L. sativum*

Identified phytoconstituents	*L. sativum*
Tannins	+++
Flavonoids	++
Triterpenoids	++
Alkaloids	+++
Saponins	++
Cardiac glycosides	−
Anthraquinones	+++

−: absent, +: low intensity, ++: medium intensity, and +++: strong intensity.

**Figure 2:**
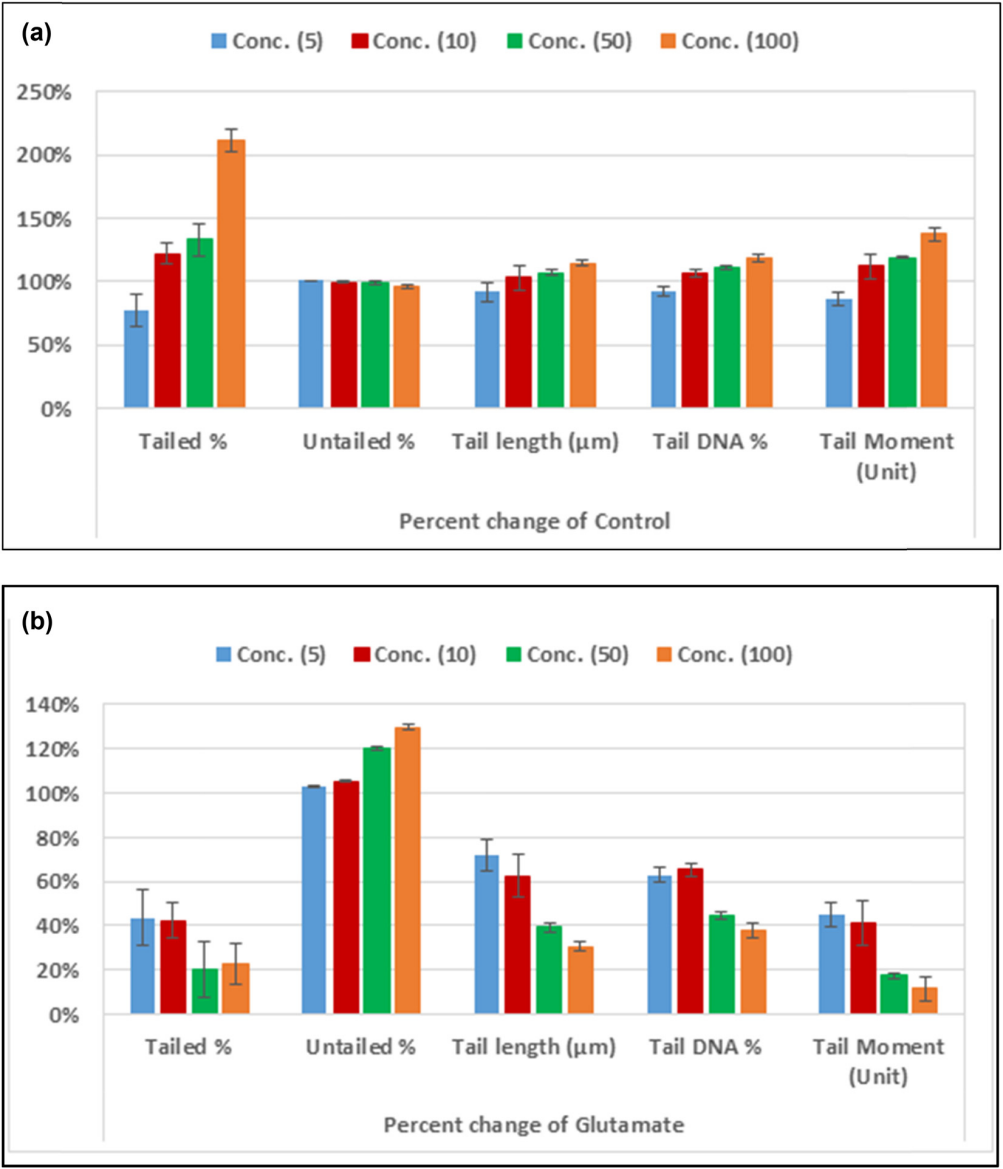
Percentage change in the five measured comet assay variables in RGCs treated with *L. sativum* compared to control healthy untreated cells (a) OR compared to glutamate excitotoxic RGCs (b).

**Figure 3:**
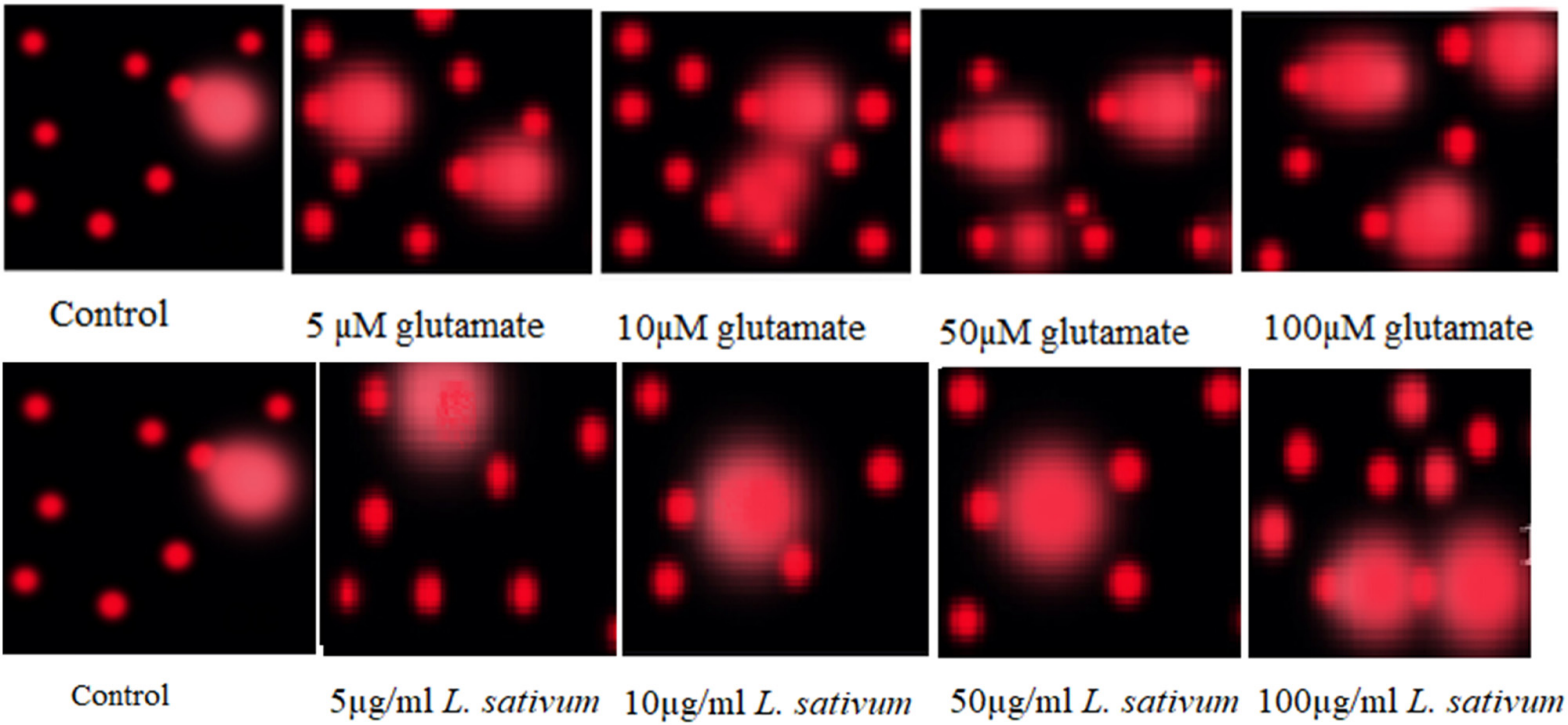
Measure of glutamate-induced DNA damage through single – cell alkaline gel electrophoresis (comet) assay in control, glutamate excitotoxic RGCs (5–100 µM glutamate), and *L. sativum*-treated RGCs (5–100 µg *L. sativum* extract).

Table 3 together with Figures 2 and 3 demonstrates that the tailed DNA (TDNA), tail length (TL), and tail moment (TM) of the 50 and 100 mM glutamate-treated groups were significantly greater than those of the blank control group (*p* < 0.05). In addition, the *L. sativum*-treated groups demonstrated nonsignificantly different tailed DNA (TDNA), tail length (TL), and tail moment (TM) when compared with the blank control group, but significantly lower values when compared with the glutamate-treated groups (*p* < 0.05 for 5 and 10 mM and *p* < 0.01 for 50 and 100 mM).

**Table 3:** Comparison COMET assay measured variables in glutamate intoxicated and plant extract-treated retinal cells

Parameters	Extracts	Concentration			
		5 µg	10 µg	50 µg	100 µg
Tailed (%)	Control	3.00 ± 1.00	3.00 ± 1.00	3.00 ± 1.00	3.00 ± 1.00
	Glutamate (5–100 µM)	5.33 ± 1.15	8.67 ± 0.58	20.00 ± 1.73	27.67 ± 2.52
	*L. sativum*	2.33 ± 0.58^b^	3.67 ± 0.58^b^	4.00 ± 1.00^b^	6.33 ± 1.15a,b
Untailed (%)	Control	97.00 ± 1.00	97.00 ± 1.00	97.00 ± 1.00	97.00 ± 1.00
	Glutamate (5–100 µM)	94.67 ± 1.15	91.33 ± 0.58	80.00 ± 1.73	72.33 ± 2.52
	*L. sativum*	97.67 ± 0.58^b^	96.33 ± 0.58^b^	96.00 ± 1.00^b^	93.67 ± 1.15a,b
Tail length (µm)	Control	1.23 ± 0.09	1.23 ± 0.09	1.23 ± 0.09	1.23 ± 0.09
	Glutamate (5–100 µM)	1.57 ± 0.13	2.03 ± 0.22	3.36 ± 0.32	4.62 ± 0.41
	*L. sativum*	1.13 ± 0.08^b^	1.27 ± 0.12^b^	1.32 ± 0.03^b^	1.41 ± 0.03^b^
Tail DNA (%)	Control	1.24 ± 0.16	1.24 ± 0.16	1.24 ± 0.16	1.24 ± 0.16
	Glutamate(5–100 µM)	1.83 ± 0.12	2.03 ± 0.11	3.06 ± 0.15^a^	3.85 ± 0.58^a^
	*L. sativum*	1.15 ± 0.04^b^	1.33 ± 0.04^b^	1.37 ± 0.02^b^	1.47 ± 0.05a,b
Tail moment (unit)	Control	1.51 ± 0.10	1.51 ± 0.10	1.51 ± 0.10	1.51 ± 0.10
	Glutamate (5–100 µM	2.88 ± 0.41	4.12 ± 0.44	10.32 ± 1.49	17.96 ± 4.09
	*L. sativum*	1.30 ± 0.07^b^	1.69 ± 0.17^b^	1.80 ± 0.02^b^	2.08 ± 0.11a,b

a There is sig. diff. with the control group at 0.05 level.

b There is sig. diff. with the glutamate group at 0.05 level and 0.01 level.

**Figure 4:**
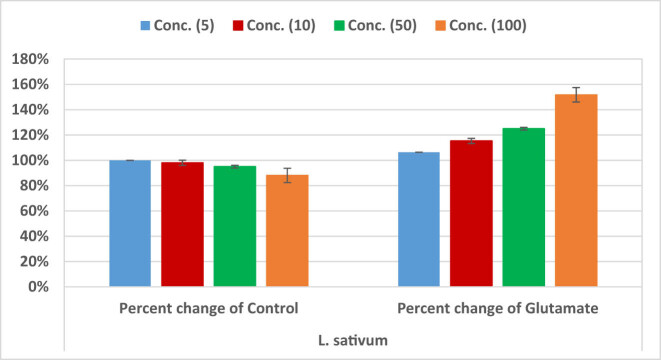
Percentage change in cell viability of RGCs – treated with different concentrations of *L. sativum* (5–100 µg) compared to healthy untreated control cells, or glutamate excitotoxic cells.

**Figure 5:**
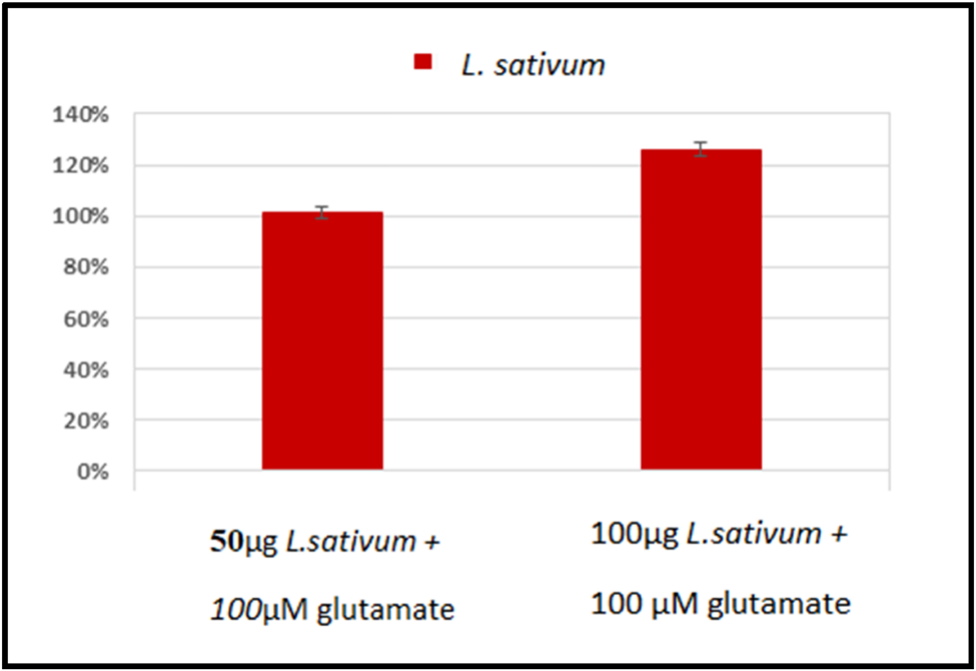
Percentage change of the neuroprotective effects of *L. sativum* (50 and 100 µg) on glutamate (100 µM) – induced loss of RGCs’ viability (Death).

Table 4 and Figure 4 show cell viability of RGCs treated with different concentrations of the excitotoxic glutamate (5–100 µM), or *L. sativum* (5–100 µg) compared to healthy untreated control cells. It can be easily noticed that while cell viability of 50 and 100 µM glutamate-treated cells recorded significantly lower cell viability of 0.76 ± 0.01 and 0.58 ± 0.03, respectively, compared to control cells, 100 µg *L. sativum*-treated cells recorded much higher cell viability 0.88 ± 0.05. The three lower concentrations of *L. sativum* did not show any cytotoxic effects on RGCs. The neuroprotective effects of *L. sativum* can be observed in Table 5 and Figure 5. Combined treatment of cells with *L. sativum* (100 µg) *+* Glutamate (100 µM) induces remarkable increase in cell viability. Table 6 demonstrates the remarkable antioxidant and scavenging effects of *L. sativum extracts.* IC_50_ of the extract was found to be concentration-dependent.

**Table 4:** Cell viability (MTT assay) of RGCs treated with different concentrations of the excitotoxic glutamate (5–100 µM), or *L. sativum* (5–100 µg) compared to healthy untreated control cells

Parameters	Extracts	Concentrations			
		5	10	50	100
Control	Control	1.00 ± 0.00	1.00 ± 0.00	1.00 ± 0.00	1.00 ± 0.00
	Glutamate (µM)	0.94 ± 0.02	0.85 ± 0.02	0.76 ± 0.01^a^	0.58 ± 0.03^a^
	*L. sativum* (µg)	1.00 ± 0.00^b^	0.98 ± 0.02^b^	0.95 ± 0.01^b^	0.88 ± 0.05a,b

a There is sig. diff. with the control group at 0.05 level.

b There is sig. diff. with the glutamate group at 0.05 level and 0.01 level.

**Table 5:** Neuroprotective effects of *L. sativum* (50 and 100 µg) on glutamate (100 µM) – induced loss of RGCs’ viability (death)

Treatment	Mean ± S.D
Glutamate (100 µM)	0.76 ± 0.01
*L. sativum* (50 µg) + Glutamate (100 µM)	0.77 ± 0.02
Glutamate (100 µM)	0.58 ± 0.03
*L. sativum* (100 µg) + Glutamate (100 µM)	0.73 ± 0.02^a^

a There is sig. diff. with the glutamate group at 0.01 level.

**Table 6:** Antioxidant activity of *L. sativum* extract

Sample	Concentration (μg/mL)	ABTS (IC_50_ μg/mL)	DPPH (IC_50_ μg/mL)
*L. stavium* seed extract	50	38.21 ± 0.37	30.24 ± 0.67
	100	26.52 ± 0.65	16.39 ± 0.82
	200	12.45 ± 3.22	6.74 ± 0.01
Butylated hydroxytoluene (BHT)	200	8.67 ± 0.98	1.26 ± 0.27

Comparing between all groups using One-Way ANOVA test with Multiple Comparisons (Dunnett test) to compare each group with the glutamate group.

## 4 Discussion

Our understanding of the etiological and biochemical mechanisms of numerous neurological disorders has immensely increased. Parallel with this, growing knowledge has been obtained that might help in the understanding of the mechanisms through which a number of naturally occurring plant extracts can affect these pathological pathways so as to offer protection against damage and support healing of neurological tissues [39].

As shown in Table 1, the results revealed that quercetin and nariginin were found in high quantity, whereas the kaempferol was recorded in low amounts. This could be easily related to the ameliorative effects of *L. sativum* extract on glutamate-induced DNA damage, cell viability, and the antioxidant effects or scavenging activity. This is in good agreement with two recent studies which demonstrated that quercetin and nariginin were shown to induce mitochondrial activity, significant membrane stabilization, and prevention of lipid peroxidation of treated cells [40,41]. Table 2 demonstrates the richness of *L. sativum* with tannins, flavonoids, alkaloids, and anthraquinones as phytochemical with well-documented therapeutic potency.

The comet assay or single-cell gel electrophoresis (SCGE) helps to define whether DNA damage-induced single-cell death (apoptosis) or cytotoxicity has occurred and to measure and compare the extent of this damage [42]. Cells embedded in agarose on a microscopic slide are lysed with detergent and high salt to form nucleoids holding supercoiled loops of DNA linked to the nuclear matrix. Electrophoresis results in structures that look like comets at alkaline pH and the intensity of the comet tail or tail length relative to the head usually reflects the number of DNA breaks [42].

Neurodegenerative diseases are a mixture of disorders of the nervous system, including the brain, spinal cord, and peripheral nerves, which have diverse etiologies [43]. Owing to the prevalence, morbidity, and mortality of different neurological disorders, they represent major social, medical, and economic issues [44]. Many environmental factors are connected to neurological disorders. Among these are food, heavy metals, chemicals, and lifestyle [44]. Food additives such as monosodium glutamate (MSG) are recognized as brain toxicants closely linked to neuronal death through glutamate-induced excitotoxicity as a major etiological mechanism in neurological disorders.

Table 3 and Figures 2 and 3 demonstrate the excitotoxic effects of glutamate on pure RGCs in culture. Dose-dependent increases of tailed%, tail length, and tail moment in glutamate-treated RGCs were observed. While low concentrations of glutamate (5 and 10 mM) did not show significant differences compared with the control, higher concentrations (50 and 100 mM) showed significant variation in the four comet assay variables as measures of DNA fragmentation. This is supported by the work of Ankarcrona et al. [45] and Culmsee et al. [46] who proved that high concentrations of glutamate (≥100 μM) can induce neuronal death, which characteristically involves DNA damage and the initiation of apoptosis in rat primary cerebral cortical cultures. Moreover, the nonsignificant findings of DNA damage with 5 and 10 mM glutamate are in good agreement with the previous work by Yang et al. [47] who reported that the exposure of neurons to a low concentration of glutamate (20 μM) for 10 min was effective to induce oxidative nuclear DNA damage, but did not cause significant cell death [48]. Based on the contribution of glutamate excitotoxicity as an etiological mechanism of many neurological disorders, the obtained data can prove that DNA damage and apoptosis are neurological signals of glutamate toxicity, which can be targeted to treat major neurodegenerative diseases such as Alzheimer’s disease [48], Parkinson’s disease [49], and retinal neurodegeneration [50].

There is no cure for many neurological disorders, but treatment of the symptoms may improve the clinical presentation of many disorders such as dementia in Alzheimer’s disease; tremor, slowness, and stiffness in Parkinson’s disease; impaired social interaction in autism spectrum disorders (ASD); and other brain-related problems. Traditional medicine has long been practiced globally in the form of using memory enhancers and medicinal plants in the treatment of dementia, amnesia, as well as Alzheimer’s disease. Most applied herbs and plants have been chemically evaluated and their efficacy has also been proven in clinical trials. However, the mechanisms underlying the activities of medicinal plants as suggested treatments for neurological disorders are still under investigation [51].

Table 3 and Figures 2 and 3 also show the ameliorative effects of *L. sativum* in glutamate-induced DNA damage measured as % tailing, tail length, and tail moment. Much lower values of these variables were observed with significant variation compared with glutamate-treated cells, but there were nonsignificant differences when compared with the blank control group of RGCs.

The reported protective effect of *L. sativum* against glutamate-induced DNA damage is supported by the recent study by Zamzami et al. [52], in which almost complete repair of DNA damage occurred posttreatment of rabbits with 200 mg and 400 mg/kg body weight *L. sativum* seeds. The protective effect of *L. sativum* seeds on hepatic DNA damage can be attributed to the antioxidant activity of *L. sativum* seeds through direct scavenging of free radicals or interfering with free radicals production.

Based on the fact that oxidative stress and detoxification mechanisms are linked to glutamate excitotoxicity as three etiological mechanisms in neurological disorders, the ameliorative effects of *L. sativum* reported in the present study can be attributed to its antioxidant and detoxification mechanisms. Through the use of multiple regression, El-Ansary [53] proved the relationship between these three mechanisms and the etiology of ASD as a neurodevelopmental disorder of remarkably increasing prevalence. They also recently suggested *L. sativum* as a complex nutritional supplement to treat ASD [54].

The glutamate NMDARs on membranes can be roughly divided into two groups: synaptic and extrasynaptic receptors. Some studies proved that extrasynaptic NMDARs are more responsible for glutamate excitotoxicity and DNA damage-induced cell death than synaptic ones [55,56,57,58]. It is widely believed that synaptic NMDAR activation stimulates cell survival, while extrasynaptic activation triggers cell death; in addition, it is thought that imbalance between the two contributes to neuronal dysfunction as a major cause of most neurological disorders [59]. For example, in Alzheimer’s disease, excess glutamate released from astrocytes activates extrasynaptic NMDARs and triggers proapoptotic signaling, which overcomes synaptic NMDAR-mediated survival signaling that is already weakened by other mechanisms such as the endocytosis of NMDARs. This leads to further synaptic damage and eventual neuronal death. Based on this, the anti-excitotoxic effect of *L. sativum* reported in the current study can help to suggest this plant as an option for treating Alzheimer’s disease [48], Parkinson’s disease [49], and retinal neurodegeneration [50].

As the retina is considered an extension of the brain, it could provide a stage for studying neurological disorders, understanding the contributing etiological mechanisms, and subsequently developing treatment strategies. Table 4 and Figure 4 demonstrate the independent effect of treating RGCs with glutamate, as an excitotoxic agent, and *L. sativum* as a promising source of a plant extract that might help in treating excitotoxicity. This table clearly shows that glutamate significantly reduced RGC viability at high concentrations (50 and 100 mM) when compared with healthy control cells. This is supported by the recent work by Christensen et al. [60], who proved the susceptibility of RGCs to glutamatergic excitotoxicity. On the other hand, the viability of *L. sativum-*treated RGCs was significantly different from that of glutamate-treated cells, but not that of control cells, especially at the concentrations of 5, 10, and 50 µg/mL. Regarding *L. sativum*, 100 µg/mL was the only concentration at which significantly lower cell viability was recorded compared with that of the control. Table 5 and Figure 5 show the ameliorative effects of *L. sativum* on glutamate-induced cell death, presenting a remarkable increase of cell viability. This is in good agreement with the previous work of Al-Sheddi et al. [61] who ascertained the cytoprotective effects of *L. sativum* extract against H_2_O_2_-induced cell death. The antioxidant effects of *L. sativum* are shown in Table 6. This could be attributed to the multiple antioxidant compounds recorded in this plant (Tables 1 and 2) which demonstrate high scavenging power of lipid peroxides and ROS [40,41].

This might be attributed to the multiple antioxidant and anti-inflammatory components of this plant, as shown in Tables 1 and 2. This is supported by the recent work of Chatoui et al. [62] who proved that the antioxidant activity of different extracts of *L. sativum* correlated significantly with their total flavonoids and other phenolic compounds. They suggested that *L. sativum* seeds could be used in food supplement preparations or as a food additive protecting against oxidative stress as a major signaling pathway related to glutamate excitotoxicity, a major etiological mechanism of almost all neurological disorders.

## 5 Conclusion

In conclusion, our study indicates the effectiveness of methanol extract of *L. sativum* in ameliorating changes in comet assay-related variables as markers of DNA damage and reduced cell viability induced in RGCs after exposure to high concentrations of glutamate. This work suggests the value of using *L. sativum* seed extracts as anti-excitotoxic agents, which might improve the clinical presentation of many neurological disorders.
